# Full-Field Temperature Measurement of Stainless Steel Specimens Subjected to Uniaxial Tensile Loading at Various Strain Rates

**DOI:** 10.3390/ma14185259

**Published:** 2021-09-13

**Authors:** Krzysztof Żaba, Tomasz Trzepieciński, Stanislav Rusz, Sandra Puchlerska, Maciej Balcerzak

**Affiliations:** 1 Department of Metal Working and Physical Metallurgy of Non-Ferrous Metals, Faculty of Non-Ferrous Metals, AGH—University of Science and Technology, al. Adama Mickiewicza 30, 30-059 Cracow, Poland; spuchler@agh.edu.pl (S.P.); maciejbalcerzak1@gmail.com (M.B.); 2 Department of Manufacturing and Production Engineering, Faculty of Mechanical Engineering and Aeronautics, Rzeszow University of Technology, al. Powst. Warszawy 8, 35-959 Rzeszów, Poland; tomtrz@prz.edu.pl; 3 Department of Mechanical Technology, Faculty of Mechanical Engineering, VŠB—Technical University of Ostrava, 17 listopadu 15, CZ 708 33 Ostrava–Poruba, Czech Republic; stanislav.rusz@vsb.cz

**Keywords:** digital image correlation, mechanical properties, stainless steel, temperature, thermovision, uniaxial tensile test

## Abstract

This article presents a study on the effect of strain rate, specimen orientation, and plastic strain on the value and distribution of the temperature of dog-bone 1 mm-thick specimens during their deformation in uniaxial tensile tests. Full-field image correlation and infrared thermography techniques were used. A titanium-stabilised austenitic 321 stainless steel was used as test materials. The dog-bone specimens used for uniaxial tensile tests were cut along the sheet metal rolling direction and three strain rates were considered: 4 × 10^−3^ s^−1^, 8 × 10^−3^ s^−1^ and 16 × 10^−3^ s^−1^. It was found that increasing the strain rate resulted in the intensification of heat generation. High-quality regression models (Ra > 0.9) developed for the austenitic 321 steel revealed that sample orientation does not play a significant role in the heat generation when the sample is plastically deformed. It was found that at the moment of formation of a necking at the highest strain rate, the maximum sample temperature increased more than four times compared to the initial temperature. A synergistic effect of the strain hardening exponent and yield stress revealed that heat is generated more rapidly towards small values of strain hardening exponent and yield stress.

## 1. Introduction

Metals and their alloys have a crystalline structure, which is characterised by a regular arrangement of atomic cores. Technical metals obtained by conventional metallurgical methods have a polycrystalline structure [1,2]. This means that metals are made up of grains characterised by approximately the correct crystal structure. Polycrystalline materials consist of grains with various orientations. For many polycrystalline materials, the grain orientations are random before any working (deformation) of the material is undertaken. Therefore, even if the individual grains are anisotropic, the property differences tend to average out and, overall, the material is isotropic. When a material is formed, the grains are usually distorted and elongated in one or more directions which makes the material anisotropic [3].

In metals subjected to plastic working, the most important defects of the crystal lattice are dislocations and grain boundaries. Strain hardening—hardening of a material with deformation—results from interaction and multiplication of dislocations during plastic deformation. Grain boundaries have a much greater influence on the mechanical properties of metals than dislocations [4,5]. The high energy level of grain boundaries determines the occurrence of many phenomena, such as continuous mobility of boundaries leading to grain growth and lower corrosion resistance [6,7,8]. Grain boundaries also have a strong influence on the ductility of metals. The degree of strain hardening of materials by grain refining is described by the Hall–Petch relationship [9,10] according to which the yield strength of the material increases with the degree of grain refinement. On the one hand, grain boundaries inhibit the free movement of dislocations during grain deformation while participating in the process of material strengthening and in the formation of the deformation texture [1]. On the other hand, the grain boundaries are the main area of permanent deformation during creep or superplastic flow.

The deformation of the material (elastic or plastic) occurs under the influence of a load applied to it. Elastic deformation disappears when the load is removed. Plastic deformation remains after the material is unloaded. There are two main mechanisms of plastic deformation: the gliding motion of dislocations and deformation twinning [11,12]. The gliding motion does not occur simultaneously in all possible planes and directions of slip. Deformation occurs gradually, starting from the sliding planes and directions prioritised in relation to the direction of the applied load. During deformation, the number of active slip planes and free dislocations that are generated during deformation increases. Further plastic deformation requires the application of greater stress in order to initiate new dislocations [13,14,15]. The process by which stress increases with deformation is called work hardening. Plastic deformation is also accompanied by heat generation due to the internal resistance of the material, which can be detected at the microstructure scale when loading the material [16,17].

The investigation of heat generation and dissipation during plastic deformation was reflected in scientific research. Bodelot et al. [18] applied infrared (IR) coupled full-field measurements to observe the heterogeneities of kinematic and thermal data at the grain scale of austenitic stainless steel. Specimens were subjected to a cyclic loading. It was found that the temperature was significantly affected by diffusion and this observation tends to confirm that temperature may not be used as a damage indicator. Boulanger et al. [19] have focused on the determination of heat sources from a temperature field provided by an IR camera. They separately identified the dissipative and thermo-elastic heat sources. Thermal full-field measurements from infrared thermography were used to study the Portevin-Le Chatelier effect [20]. The temperature field provided insights into the dynamics of band formation and motion.

The non-contact optical methods for the measurement of temperature displacement include video extensometers [21], laser speckle correlation [22], interferometry methods [23,24,25] and temperature-calibrated CCD cameras [26]. In addition to IR thermography, the other technique that allows temperature changes to be coupled with strains is digital image correlation (DIC), in which digital images of an object before and after deformation are captured using a non-contact optic and material-independent measuring instrument, and then they are subject to correlation analysis [23,24,27]. High-speed DIC allows deformation and damage mechanisms to be analysed in a quantitative manner [28]. DIC was used to obtain strain fields and to investigate the propagation of the Lüders band in steel specimens subjected to the uniaxial tensile test [29]. The strain states associated with localised necking and diffuse strains are also investigated. Results show that the cross-section of a tensile specimen must be regarded as a structure, not as an elementary volume of material which is subjected to the uniform load. The results of investigations of Hung and Voloshin [30] indicated that for the uniform tension test, DIC is a very convenient and efficient tool for the measurement of in-plane strain measurement. Feng and Xue [31] applied infrared thermography tests and DIC tests for the display of the thermal field during mechanical tensile tests of 3D printed bolts. Cholewa et al. [32] developed the method of calibrated infrared thermal cameras with a stereo-vision DIC system applicable to scales of lengths both large and small. Żaba et al. [33] used DIC and IR thermography to find the relationship between yield stress and the Taylor–Quinney coefficient and their change with the strain rate during the stretching of nickel-based superalloys. A coupled thermography and DIC system was calibrated using a series of one sided heat exposure experiments performed on loaded sandwich composites. Infrared thermography and DIC measurements are not applicable to experiments with low conductivity materials or discontinuous materials [33,34]. Maynadier, et al. [35] developed an infrared image correlation system equipped with a single IR camera to measure simultaneous thermal fields and deformation. The disadvantage of the proposed method is that this technique requires the use of a special coating for displacement measurement using the single camera. Chrysochoos et al. [34] proposed DIC and thermography measurements for the determination of the mechanical energy and heat sources involved at local scale during a heterogeneous tensile test. Thermodynamic analysis of the energy balance showed the influence of the dissipated mechanism on the stress–strain response.

Many of studies take place at a macroscopic scale with the measurement of the average temperature of the material [36,37]. Meanwhile, due to the grain structure of polycrystalline metals and the occurrence of various plastic deformation mechanisms, the temperature of the body subjected to loading is not uniform. The way to explain this phenomenon is to take full-field measurements. In this paper, the coupled IR and DIC measurements were conducted to assess the thermomechanical response of stainless steel strip samples subjected to tensile load at different strain rates. An analysis of variance (ANOVA) was used to gain information about the relationship between the strain hardening phenomenon, yield stress, sample orientation in respect of the sheet rolling direction, strain rate, and plastic strain of the specimen and temperature.

## 2. Materials and Methods

### 2.1. Material

**Figure 1:**
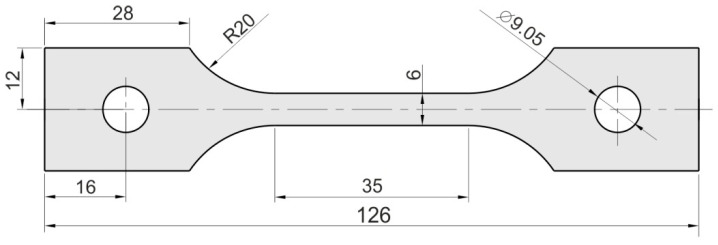
Dimensions (in mm) of the samples for the tensile test.

The research material was a titanium-stabilised austenitic stainless steel, 321 (AMS 5510). Steel 321 is a steel with good formability, weldability and resistance to intergranular corrosion that is mainly used in the chemical and aviation industries. The thickness of the sheets was 1 mm. The test samples were cut from the sheet in three directions: the rolling direction (RD), at an angle of 45° to the RD and perpendicular to the RD. The dimensions of test samples (Figure 1) were in accordance with the ASTM E8/E8M–11 standard [38]. The requirements for the chemical composition of the test steel are shown in Table 1 and are in accordance SAE AMS 5510 [39].

### 2.2. Uniaxial Tensile Test

**Figure 2:**
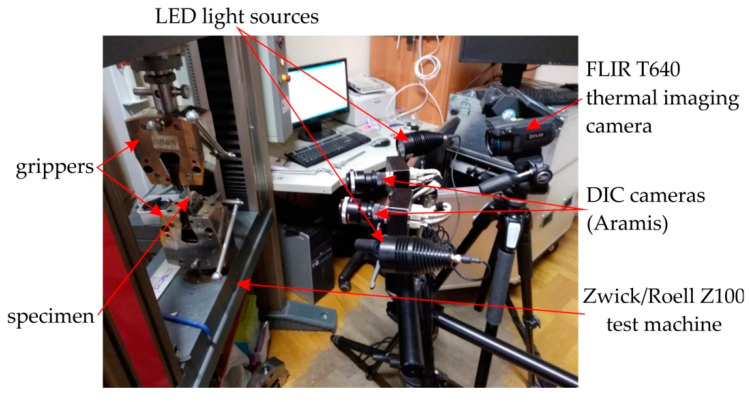
Test stand.

Dog-bone samples (Figure 1) were stretched in a uniaxial tensile test machine Zwick/Roell Z100 (Figure 2). Three different strain rates were used in the investigations: 4 × 10^−^^3^ s^−^^1^, 8 × 10^−^^3^ s^−^^1^ and 16 × 10^−^^3^ s^−^^1^. The tests were carried out at a temperature of 24 °C.

### 2.3. Digital Image Correlation Technique

The digital image correlation Aramis system (GOM, Braunschweig, Germany) was used to determine the character of the deformation of the sample in a non-contact manner during the stretching process of samples. The measuring system consists of two essential components. The first is a scanner, consisting of two high-resolution digital cameras (Figure 2) positioned in relation to each other in such a way as to be able to build a spatial image. The second element of the set is a computer with special software for the numerical processing of images.

Proper surface preparation of the samples is necessary for DIC analysis. First, the samples are covered with white paint, and then a random pattern of contrasting black spots is applied. The selection of the size of the spots depends on the size of the test object and the optical properties of the measuring apparatus. The measurement process consists in taking a series of images of the sample by two cameras in successive stages of loading. Correlation of images from these cameras makes it possible to determine the position of each pixel by giving them coordinates in a three-dimensional coordinate system. Then the images are divided into so-called deformation grids, each of which contains a unique pattern of dots [40]. The initial setup becomes the reference step. Due to the deformation of the sheet surface, the spots in each element of the mesh change their position in relation to each other, which makes it possible to calculate the full field deformation in relation to the reference position. However, in order to make such an analysis possible, the system divides the measurement area into fields of a fixed size called facets. Local strains are obtained from the formula:

(1) $$\varepsilon_{eng}=\left(\underset{l\to 0}{\lim}\left(\frac{l_{in}+\Delta l_{d}}{l_{in}}\right)-1\right)\cdot 100\%$$

where *l_in_* is the initial dostance between two neighbouring facets and Δ*l_d_* is the distance increase during uniaxial tensile test.

The dimension of standard facet is 21 × 21 pixels. The second characteristic value is the distance between the centres of adjacent facets. The Aramis system offers many benefits, such as:

- a stable solution for full-field analyses of test objects of just a few millimeters up to structural components of several metres in size,
- it is a material-independent and non-contact measuring system,
- it performs high-precision measurements with a 3D measurement resolution in the sub-micrometer range,
- it is a high-resolution for point-based and full-field measurements.

### 2.4. Infrared (IR) Thermal Mapping

The surface temperature of the samples during the stretching was measured using a high-sensitivity IR Flir T640 camera (Flir Systems AB, Antennvägen 6, 187 66 Täby, Sweden). The measurements of temperature were correlated with the measurement of strain using the A0Aramis system. Non-invasive distance measurement was performed with an accuracy of thermographic measurements in the range of +/− 2 °C. The principal parameters of the Flir T640 camera are listed in Table 2.

### 2.5. Analysis of Variance

Quadratic multidimensional ANOVA was used to determine the relationship between material properties (strain hardening exponent, yield stress, sample orientation), process parameters (strain rate and percentage strain) and the maximum temperature appearing in the sample during the stretching process. The values of the strain hardening exponent were determined by approximating true stress-true strain curves using the well known Hollomon’s power law.

ANOVA is a statistical method for examining observations that depend on one or more factors acting simultaneously. Due to the different numerical ranges of the data, they were normalised to the range [−1, +1] [41]. The *min*-*max* normalisation was applied by means of a linear function bringing the data to a new interval (Coded Low, Coded High).

Explanatory variables should be independent of each other. The input data were fitted with a polynomial, and the influence of individual variables on the quality of the model was checked using backward elimination. The minimum and maximum values of the input variables for samples made of 321 sheet are presented in Table 3. Intermediate values of input parameters were coded proportionally in the range [−1, +1].

The regression models that were built were subjected to significance tests. The method of backward elimination of variables was used in the analysis. It is a variable selection procedure in which all the variables are entered into the regression equation and then removed sequentially. The variable with the lowest partial correlation with the dependent variable is considered for removal first. If it meets the elimination criteria, it is removed. The basis for removing or leaving a given variable in the model is the calculation of Fisher F statistics. The independent variable with the highest probability corresponding to the Fisher parameter F is removed from the model if the probability *p* is sufficiently high (typically *p* = 0.10). After the first variable is removed, the next one to be removed is the one that has the smallest partial correlation with the dependent variable. The procedure exits when there are no other variables in the equation that meet the removal criteria. The significance of the regression model at a level of α = 0.05 is determined based on the variance due to the effect of a factor and the variance due to the error term.

## 3. Results and Discussion

### 3.1. Experimental Investigations

**Figure 3:**
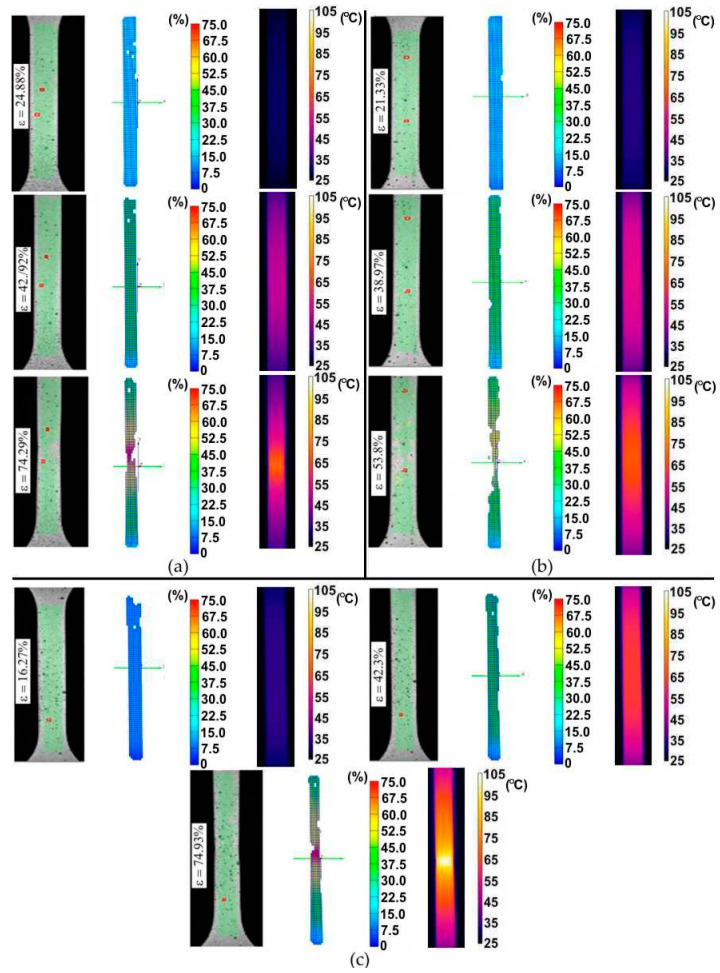
Infrared (IR) thermography images and digital image correlation charts for 321 steel samples cut along the sheet rolling direction, tested at the following strain rates: (**a**) 4 × 10^−^^3^ s^−^^1^, (**b**) 8 × 10^−^^3^ s^−^^1^ and (**c**) 16 × 10^−^^3^ s^−^^1^.

**Figure 4:**
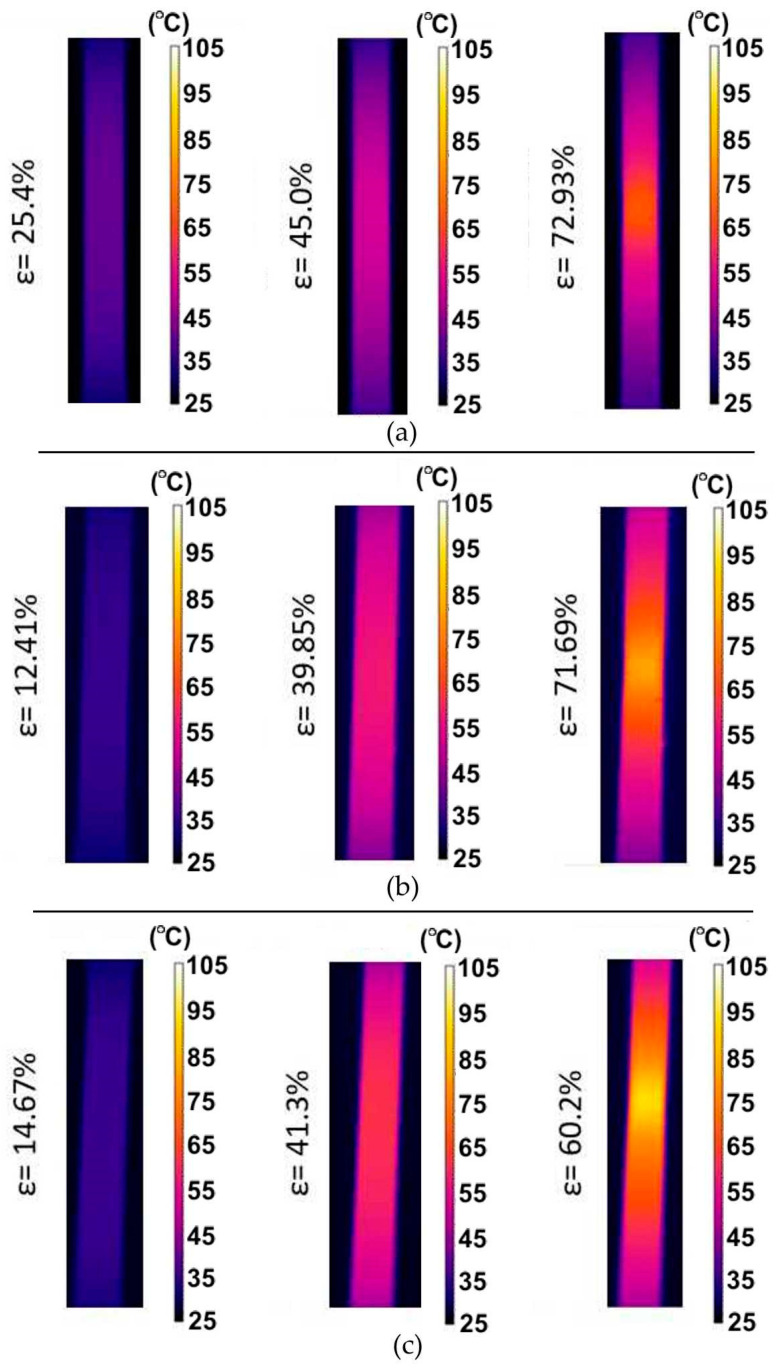
IR thermography images and digital image correlation charts for 321 steel samples cut perpendicular to the rolling direction (RD), tested at the following strain rates: (**a**) 4 × 10^−^^3^ s^−^^1^, (**b**) 8 × 10^−^^3^ s^−^^1^ and (**c**) 16 × 10^−^^3^ s^−^^1^.

Figure 3 shows the evolution of the surface temperature of the samples cut along the sheet rolling direction of the 321 steel during its stretching with different strain rates. It is clearly visible that even within the range of proportional deformations the zone with the highest temperature is located approximately at the central point between the grippers of the testing machine. As a result of heat convection caused by internal friction of the material, an increase in the temperature of the samples was also observed in the part that is gripped where no plastic deformation occurs. Increasing the strain rate resulted in the intensification of heat generation. At the moment of the formation of a necking at the highest strain rate, the maximum sample temperature increased by more than 4 times compared to the initial temperature. Similar conclusions can be drawn for the samples cut at an angle of 45° with respect to RD and perpendicular to RD (Figure 4).

Only part of the mechanical energy is converted into heat in the deformation process. The remainder is stored in the microstructure of the material increasing the internal energy of the material. The total energy spent on deforming an elastic-plastic material is equal to the work undertaken on elastic (reversible) deformation and permanent (plastic) deformation. Moreover, the energy consumed on plastic deformation is divided into heat dissipated in the forming process and energy stored in the material [42,43,44]. The evolution of the microstructure during deformation depends on the type of material, the initial temperature and the loading situation [45,46]. Changing the deformation method usually leads to a partial or complete reconstruction of the dislocation systems formed in the previous stages of deformation and the formation of new systems [47,48]. The interaction of the lattice defects is related to the overlapping of their stress fields. If the overlap of stress fields caused by defects reduces the energy of the system, then with a specific activity of the respective slip systems, these defects will form configurations compliant with the principle of energy minimisation [49,50].

The plasticity margin measured as the difference between ultimate tensile stress and field stress is more pronounced in the case of 321 steel than for 17-4PH steel.

**Figure 5:**
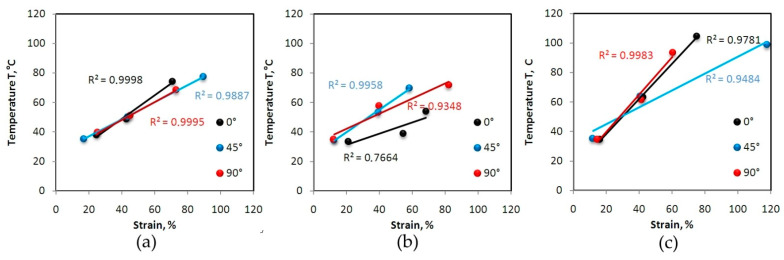
Influence of strain on temperature for 321 steel samples tested at the following strain rates: (**a**) 4 × 10^−^^3^ s^−^^1^, (**b**) 8 × 10^−^^3^ s^−^^1^ and (**c**) 16 × 10^−^^3^ s^−^^1^.

**Figure 6:**
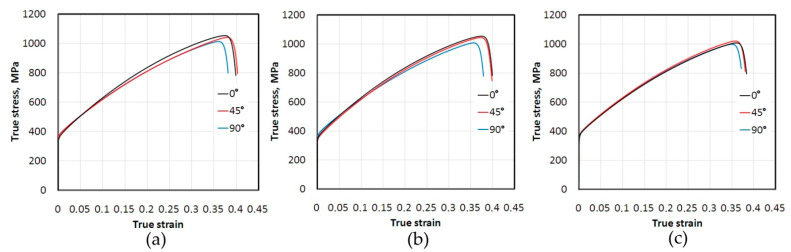
True stress-true strain curves for 321 steel determined at the following strain rates: (**a**) 4 × 10^−^^3^ s^−^^1^, (**b**) 8 × 10^−^^3^ s^−^^1^ and (**c**) 16 × 10^−^^3^ s^−^^1^.

Figure 5 show the effect of percentage strain on the change in the maximum temperature of the specimens of 321 steel. These dependencies, determined by the determination coefficient R^2^, show a linear trend with a high correlation value R^2^ > 0.93. In the case of the sample made from 321 steel cut at an angle of 45° tested at a strain rate of 16 × 10^−^^3^ s^−^^1^, the last point corresponds to the advanced stage of sample necking. Therefore, the R^2^-value for that case is about 0.9484 (Figure 5c). In general, the higher the strain rate the steeper the trend lines. The samples made of 321 steel show anisotropic features, which are particularly visible at a strain rate of 8 × 10^−^^3^ s^−^^1^ (Figure 5b). Trend lines for samples cut at different angles are inclined at different angles with respect to the abscissa axis. True stress-strain curves for 321 steel determined at various strain rates are shown in Figure 6. True strain is defined as *ε_t_*:

(2) $$\varepsilon_{t}=ln\left(\frac{l}{l_{0}}\right)$$

where *l* and *l*_0_ are the current and initial gauge lengths, respectively.

### 3.2. Analysis of Variance (ANOVA) for 321 Steel Samples

A quadratic model containing only statistically significant elements was chosen to fit the experimental data. The temperature response surface for the 321 steel specimens is given by:

(3) $$T=76.71-18.56A-10.02B-4.19C+4.71D+48.61E-4.97AB\;+9.58AC+8.27AD-31.41AE-12.99BC-13.1BE\;-21.35CE-1.22DE+13.36E^{2}$$

The significances of the influence of the individual material parameters as well as the conditions of the stretching process and their interaction in the regression model were determined on the basis of the ANOVA. The quality of fit of the model obtained to the values measured for a given research plan (Equation (3)) was then determined on the basis of the determination coefficients R^2^ and the F test. The significance of the model is confirmed by an F-value of 53.30 (Table 4). This model can, therefore, be used to predict temperature values based on material deformation. The probability that such a large F-value can result from data noise is only 0.01%.

The probability values *p* for *E*, *AE*, *CE*, *E*^2^ that are less than 0.0500 prove that these factors are statistically significant. The parameters the removal of which will not reduce the quality of the model include, inter alia, *C*, *AB*, *BC*, *DE*. Due to the satisfactory value of the determination coefficient for the model equal to R^2^ = 0.9868 (Table 5), it was decided to leave these variables in the model. The predicted value of the coefficient of determination R^2^ = 0.8982 is in reasonable agreement with the adjusted coefficient of determination R^2^ = 0.9683 because the difference between their values is less than 0.2 [51]. The value of adequacy precision 27.6342 is greater than 4. The adequacy of model is, therefore, confirmed.

**Figure 7:**
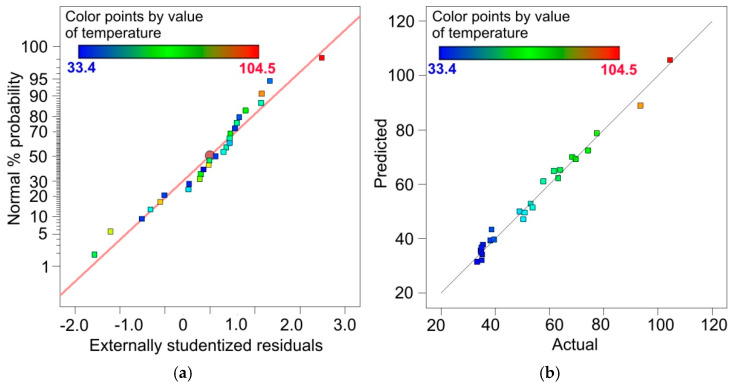
(**a**) Normal % probability vs. externally studentised residuals and (**b**) predicted vs. actual values of temperature plots for 321 steel samples.

If the errors arising during the experimental tests are random, then the distribution of residuals in the form of deviations between the actual values and the expected values should be in accordance with the normal distribution. The distribution of residuals along a straight line (Figure 7a) shows that the studentised residuals have a normal distribution [52]. Figure 7b shows a comparison of the actual values and the predicted temperature values resulting from the introduction of independent variables to the regression model. The distribution of data around a straight line indicates that the regression model is of good quality [53,54].

**Figure 8:**
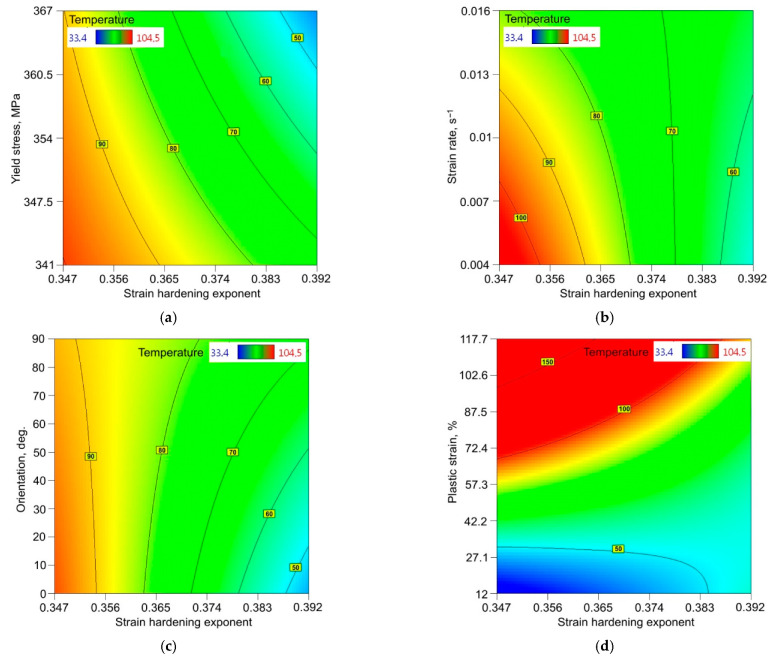
Response surface plots presenting the interaction between the strain hardening exponent and (**a**) yield stress, (**b**) strain rate, (**c**) sample orientation and (**d**) plastic strain, affecting the value of the maximum temperature of the surface of the 321 steel sample.

**Figure 9:**
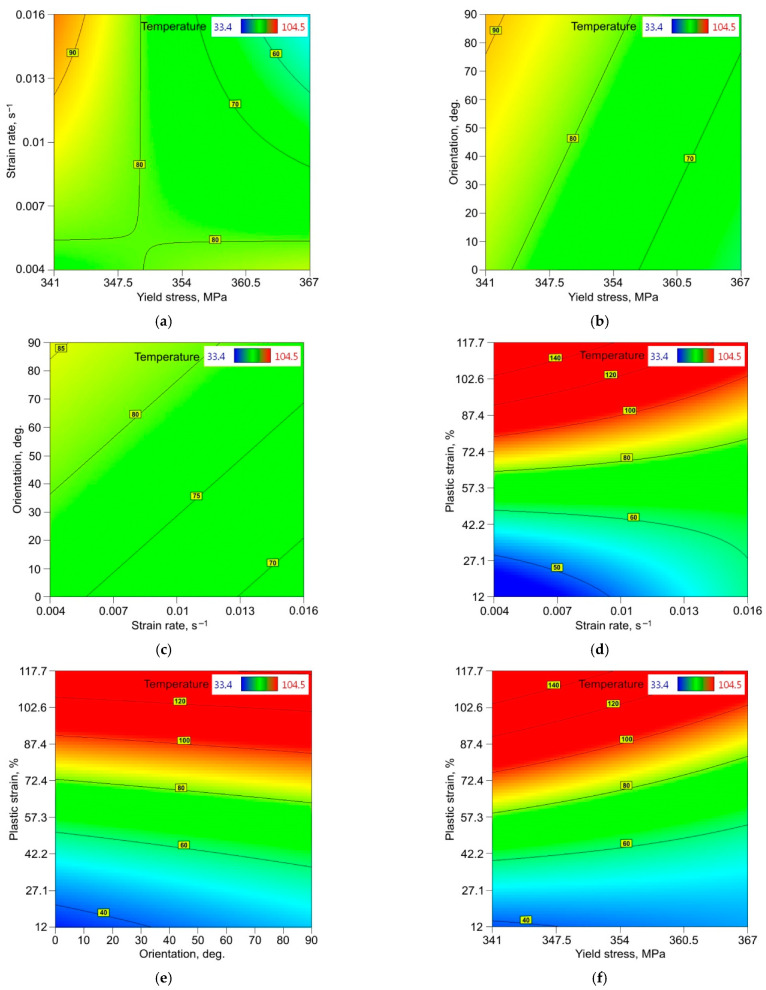
Response surface plots presenting the interaction between (**a**) strain rate (SR) and yield stress (YS), (**b**) sample orientation and YS, (**c**) sample orientation and SR, (**d**) plastic strain (PS) and SR, (**e**) PS and sample orientation and (**f**) PS and YS, affecting the value of maximum temperature of 321 steel sample surface.

The relationship between the dependent and independent variables was analysed using 2D response surface plots. Figure 8 and Figure 9 show the interactive effect of two selected parameters on the value of the maximum temperature in the sample during the stretching test when the other input parameters are constant [55,56,57]. Figure 8a indicates the synergistic role of the strain hardening exponent (SHE) and yield stress (YS). The heat is generated faster towards the low values of both the SHE and YS. Figure 8b indicates that the SHE plays a more significantly influential role in heat generation. However, the strain rate (SR) is a significant factor, but only when considering small values of SHE. For small values of SHE, the sample orientation in interaction with the SHE does not significantly affect the temperature value (Figure 8c). However, if the SHE increases then the difference between the temperature for 0° and 90° orientations increases to around 30 °C. For small values of SHE, starting from a plastic strain (PS) of about 80%, the regression model overestimates the temperature values (Figure 8d). However, the trend observed in the experimental studies is maintained, i.e., with an increase in PS for a given SHE value, the temperature value increases.

The synergistic effect of both SR and YS is more complicated (Figure 9a). Similarly, in the case of sample orientation and YS (Figure 9b), it is only for the lowest value of YS that strain rate and orientation significantly affect the value of temperature. Figure 9c indicates that the orientation when testing samples with different SR values plays the following role: for orientation 0°, the temperature is low and increases with change in the orientation to 45° and 90°. The effect of PS and SR shown in Figure 9d is very similar to the effect of PS and SHE on temperature (Figure 8d). A strong correlation between PS and temperature is also confirmed in Figure 9e,f by the high isotherm concentration. Specimens cut at 90° heat faster during deformation than specimens cut at 0° (Figure 9e). The reverse effect is observed in Figure 9f: when the YS of specimens is small, the material heats up faster.

## 4. Conclusions

In this paper, full-field image correlation and infrared thermography techniques were used to analyse the effect of material properties and strain rate on heat generation in stretched dog-bone specimens of titanium-stabilised austenitic stainless steel. Based on the experimental results and analysis of variance, the following conclusions can be drawn:

- The heat during stretching of the samples was not generated proportionally over the entire length of the samples. In the range of proportional deformation (before necking formation), the zone with the highest temperature was located approximately centrally along the distance between the grippers of the testing machine.
- At the moment of formation of a necking at the highest strain rate, the maximum sample temperature increased more than four times compared to the initial temperature. This phenomenon was observed for all sample orientations analysed.
- The character of the effect of plastic strain on the maximum temperature generated in the specimens was close to a linear trend in the range of strain rates analysed.
- A synergistic effect of the SHE and YS revealed that heat was generated more rapidly towards small values of SHE and YS.
- For small values of SHE, the sample orientation in interaction with the SHE did not significantly affect the temperature value.
- For all the orientations analysed, the temperature increased in a similar way with increasing PS.

## Figures and Tables

**Table 1:** Chemical composition of 321 steel (wt.%) [39].

C (Max.)	Si	Mo	Mn (Max.)	P + S (Max.)	Cr	Ni	Ti (Max.)	N (Max.)	Fe
0.08	0.25–1.00	0.75	2.0	0.04 P 0.03 S	17.0–19.0	9.0–12.0	0.7	0.1	balance

**Table 2:** Parameters of a Flir T640 camera.

Parameter	Unit	Value
Resolution	pixel	2048 × 1536
Measuring range	°C	−40 ÷ +2000
Refresh rate	Hz	30
Thermal sensitivity	mK	30

**Table 3:** Factors and levels for analysis of variance (ANOVA) of stretching of specimens of 321 steel.

Parameter	Name	Unit	Type	Minimum	Maximum
*A*	Strain hardening exponent	-	Numeric	0.35	0.392
*B*	Yield stress	MPa	Numeric	341	367
*C*	Strain rate	s^−1^	Numeric	4 × 10^−^^3^	16 × 10^−^^3^
*D*	Sample orientation	deg.	Numeric	0	90
*E*	Percentage strain	%	Numeric	12	117.7

**Table 4:** Results of analysis of variance for the regression model for 321 steel samples.

Source	Sum of Squares	Degrees of Freedom	Mean Square	F-Value	*p*-Value	Meaning
Model	8991.31	14	642.24	53.30	<0.0001	significant
*A*—strain hardening exponent	26.04	1	26.04	2.16	0.1723	-
*B*—yield stress	39.77	1	39.77	3.30	0.0993	-
*C*—strain rate	0.5708	1	0.5708	0.0474	0.8321	-
*D*—sample orientation	13.38	1	13.38	1.11	0.3167	-
*E*—plastic strain	989.76	1	989.76	82.14	<0.0001	-
*AB*	1.11	1	1.11	0.0920	0.7679	-
*AC*	9.23	1	9.23	0.7664	0.4019	-
*AD*	22.41	1	22.41	1.86	0.2025	-
*AE*	171.00	1	171.00	14.19	0.0037	-
*BC*	4.45	1	4.45	0.3696	0.5568	-
*BE*	36.85	1	36.85	3.06	0.1109	-
*CE*	758.38	1	758.38	62.94	<0.0001	-
*DE*	2.40	1	2.40	0.1992	0.6649	-
*E* ^2^	66.37	1	66.37	5.51	0.0409	-
Residual	120.49	10	12.05	-	-	-
Cor Total	9111.80	24	-	-	-	-

**Table 5:** Fit statistics of the regression model for 321 steel samples.

Standard Deviation	3.47	R^2^	0.9868
Mean	54.09	Adjusted R^2^	0.9683
Coefficient of variation, %	6.42	Predicted R^2^	0.8982
-	-	Adequacy precision	27.6342

## Data Availability

The data presented in this study are available on request from the corresponding author.

## References

[1] Pater Z., Samołyk G. (2013). Podstawy Technologii Obróbki Plastycznej Metali.

[2] Di Gioacchino F., Edwards T.E.J., Wells G.N., Clegg W.J. (2020). A new mechanism of strain transfer in polycrystals. Sci. Rep..

[3] Physics on Nondestructive Evaluation. https://www.nde-ed.org/Physics/Materials/Structure/anisotropy.xhtml.

[4] Yang G., Park S.J. (2019). Deformation of single crystals, polycrystalline materials, and thin films: A review. Materials.

[5] Venkataraman A., Sangid M.D. (2019). Deformation Mechanisms at Grain Boundaries in Polycrystals.

[6] Luo H., Sheng H., Zhang H., Wang F., Fan J., Du J., Liu J.P., Szlufarska I. (2019). Plasticity without dislocations in a polycrystalline intermetallic. Nat. Commun..

[7] Bahrami A., Taheri P. (2019). A study on the failure of AISI 304 stainless steel tubes in a gas heater unit. Metals.

[8] Bansod A.V., Patil A.P., Moon A.P., Khobragade N.N. (2016). Intergranular corrosion behavior of low-nickel and 304 austenitic stainless steels. J. Mater. Eng. Perform..

[9] Hall E.O. (1951). The deformation and ageing of mild steel: III discussion of results. Proc. Phys. Soc..

[10] Petch N.J. (1953). The cleavage strength of polycrystals. J. Iron Steel Inst..

[11] Roa J.J., Wheeler J.M., Trifonov T., Fargas G., Mateo A., Michler J., Jiménez-Piqué E. (2015). Deformation of polycrystalline TRIP stainless steel micropillars. Mater. Sci. Eng. A.

[12] Di Gioacchino F., da Fonseca J.Q. (2015). An experimental study of the polycrystalline plasticity of austenitic stainless steel. Int. J. Plast..

[13] Cheong K.S., Busso E.P. (2004). Discrete dislocation density modelling of single phase FCC polycrystal aggregates. Acta Mater..

[14] Ichiyanagi K., Takagi S., Kawai N., Fukaya R., Nozawa S., Nakamura K.G., Liss K.D., Kimura M., Adachi S.I. (2019). Microstructural deformation process of shock-compressed polycrystalline aluminum. Sci. Rep..

[15] Korsunsky A.M. (2009). Crystal plasticity and hardening: A dislocation dynamics study. Procedia Eng..

[16] Farren W.S., Taylor G.I. (1925). The heat developped during plastic extension of metal. Proc. R. Soc. A.

[17] Taylor G.I., Quinney H. (1933). The latent energy remaining in a metal after cold working. Proc. R. Soc. A.

[18] Bodelot L., Sabatier L., Charkaluk E., Dufrenoy P. Optical and infrared coupled full-field measurements at a mesoscopic scale. Proceedings of the 9th International Conference on Quantitative InfraRed Thermography.

[19] Boulanger T., Chrysochoos A., Mabru C., Galtier A. (2004). Calorimetric analysis of dissipative and thermoelastic effects associated with the fatigue behavior of steels. Int. J. Fatigue.

[20] Ait-Amokhtar H., Fressengeas C., Boudrahem S. (2008). The dynamics of Portevin-Le Chatelier bands in an Al-Mg alloy from infrared thermography. Mater. Sci. Eng. A.

[21] Völkl R., Fischer B. (2004). Mechanical testing of ultra-high temperature alloys. Exp. Mech..

[22] Anwander M., Zagar B.G., Weiss B., Weiss H. (2000). Noncontacting strain measurements at high temperatures by the digital laser speckle technique. Exp. Mech..

[23] Pan B., Wu D., Gao J. (2013). High-temperature strain measurement using active imaging digital image correlation and infrared radiation heating. J. Strain. Anal. Eng. Des..

[24] Pan B., Wu D., Wang Z., Xia Y. (2011). High-temperature digital image correlation method for full-field deformation measurement at 1200 °C. Meas. Sci. Technol..

[25] Sharpe W.N. (2009). A high-frequency high-temperature optical strain/displacement gage. Exp. Mech..

[26] Orteu J.J., Rotrou Y., Sentenac T., Robert L. (2007). An innovative method for 3-D shape, strain and temperature full-field measurement using a single type of camera: Principle and preliminary results. Exp. Mech..

[27] Tung S.H., Sui C.H. (2010). Application of digital-image-correlation techniques in analysing cracked cylindrical pipes. Sadhana.

[28] Hild F., Bouterf A., Forquin P., Roux S., Tsuji K. (2018). On the use of digital image correlation for the analysis of the dynamic behavior of materials. The Micro-World Observed by Ultra High-Speed Cameras.

[29] Wattrisse B., Chrysochoos A., Muracciole J.-M., Nemoz-Gaillard M. (2001). Kinematic manifestations of localisation phenomena in steels by digital image correlation. Eur. J. Mech. A Solids.

[30] Hung P.C., Voloshin A.S. (2003). In-plane strain measurement by Digital Image Correlation. J. Braz. Soc. Mech. Sci. Eng..

[31] Feng X., Xue F. (2020). Characterization of 3D printed bolts based on digital image correlation and infrared thermography. Mater. Des..

[32] Cholewa N., Summers P.T., Feih S., Mouritz A.P., Lattimer B.Y., Case S.W. (2016). A technique for coupled thermomechanical response measurement using infrared Thermography and Digital Image Correlation (TDIC). Exp. Mech..

[33] Żaba K., Trzepieciński T., Puchlerska S., Noga P., Balcerzak M. (2021). Coupled thermomechanical response measurement of deformation of nickel-based superalloys using full-field digital image correlation and infrared thermography. Materials.

[34] Chrysochoos A., Huon V., Jourdan F., Muracciole J.M., Peyrux R., Wattrisse B. (2010). Use of full-field digital image correlation and infrared thermography measurements for the thermomechanical analysis of material behaviour. Strain.

[35] Maynadier A., Poncelet M., Lavernhe-Taillard K., Roux S. (2011). One-shot measurement of thermal and kinematic fields: Infrared image correlation (IRIC). Exp. Mech..

[36] Curà F., Curti G., Sesana R. (2005). A new iteration method for the thermographic determination of fatigue limit in steels. Int. J. Fatigue.

[37] Doudard C., Calloch S., Cugy P., Galtier A., Hild F. (2005). A probabilistic two-scale model for high-cycle fatigue life predictions. Fatigue Fract. Eng. Mater. Struct..

[38] ASTM International (2011). ASTM International. ASTM E8/E8M-11. Standard test methods for tension testing of metallic materials. ASTM Book of Standards.

[39] SAE International (1991). AMS 5604-Steel, Corrosion and Heat Resistant, Sheet, Strip, and Plate 18Cr-10.5Ni-0.40Ti (SAE 30321) Solution Heat Treated.

[40] Zielecki W., Kubit A., Święch Ł. (2013). Analiza Doświadczalna Odkształcenia Elementów Klejonych w Próbie Statycznego Oddzierania. Pomiary Autom. Robot..

[41] Trzepieciński T., Szpunar M., Kaščák Ľ. (2021). Modeling of friction phenomena of TI-6AL-4V sheets based on backward elimination regression and multi-layer artificial neural networks. Materials.

[42] Brennhaugen D.D.E., Georgarakis K., Yokoyama Y., Nakayama K.S., Arnberg L., Aune R.E. (2018). Probing heat generation during tensile plastic deformation of a bulk metallic glass at cryogenic temperature. Sci. Rep..

[43] Kostina A., Iziumowa A., Plekhov O. (2014). energy dissipation and storage in iron under plastic deformation (experimental study and numerical simulation). Frat. Ed. Integrità Strutt..

[44] Rusinek A., Klepaczko J.R. (2009). Experiments on heat generated during plastic deformation and stored energy for TRIP steels. Mater. Des..

[45] Klitschke S., Trondl A., Huberth F., Liewald M. (2018). Adiabatic heating under various loading situations and strain rates for advanced high-strength steels. IOP Conf. Ser. Mater. Sci. Eng..

[46] Sada N.B. (2005). The plastic depth of heat treatment steel alloy (AISI 01) due to torsion test. J. Appl. Sci..

[47] Shen Y.F., Li X.X., Sun X., Wang Y.D., Zuo L. (2012). Twinning and martensite in a 304 austenitic stainless steel. Mater. Sci. Eng. A.

[48] Song G.S., Ji K.S., Song H.W., Zhang S.H. (2019). Microstructure transformation and twinning mechanism of 304 stainless steel tube during hydraulic bulging. Mater. Res. Express.

[49] Rubtsov V.E., Kolubaev A.V. (2009). Effect of heat generation due to plastic deformation on behavior of surface-layer material during sliding. J. Frict. Wear.

[50] Maj M. (2007). Wpływ Kierunku Wstępnego Odkształcenia na Proces Magazynowania Energii w Polikryształach. Ph.D. Thesis.

[51] Montgomery D.C. (2013). Design and Analysis of Experiments.

[52] Sen A., Srivastava M. (1990). Regression Analysis Theory, Methods, and Applications.

[53] Montgomery D.C., Peck E.A., Vining G.G. (2012). Introduction to Linear Regression Analysis.

[54] Alexopoulos E.C. (2010). Introduction to multivariate regression analysis. Hippokratia.

[55] Chauhan S.R., Dass K. (2013). Dry sliding wear behaviour of titanium (grade 5) alloy by using response surface methodology. Adv. Tribol..

[56] Khalatbari H. (2012). Investigation of Formability of Material in Incremental Sheet Metal Forming Process. Master’s Thesis.

[57] Venugopal V., Kumar K.J., Muralidharan S., Parasuraman S., Raj P.V., Kumar K.V. (2016). Optimization and in-vivo evaluation of isradipine nanoparticles using Box-Behnken design surface response methodology. OpenNano.

